# Assessment of the potential for resistance to antimicrobial violet-blue light in *Staphylococcus aureus*

**DOI:** 10.1186/s13756-017-0261-5

**Published:** 2017-09-29

**Authors:** Rachael M. Tomb, Michelle Maclean, John E. Coia, Scott J. MacGregor, John G. Anderson

**Affiliations:** 1 The Robertson Trust Laboratory for Electronic Sterilisation Technologies (ROLEST), Department of Electronic & Electrical Engineering, University of Strathclyde, Glasgow, UK; 2 Department of Biomedical Engineering, University of Strathclyde, Glasgow, UK; 3 Department of Clinical Microbiology, Glasgow Royal Infirmary, Glasgow, UK

**Keywords:** 405 nm light, Bacterial tolerance, Bacterial resistance, *Staphylococcus aureus*, EMRSA-15

## Abstract

**Background:**

Antimicrobial violet-blue light in the region of 405 nm is emerging as an alternative technology for hospital decontamination and clinical treatment. The mechanism of action is the excitation of endogenous porphyrins within exposed microorganisms, resulting in ROS generation, oxidative damage and cell death. Although resistance to 405 nm light is not thought likely, little evidence has been published to support this. This study was designed to establish if there is potential for tolerance development, using the nosocomial pathogen *Staphylococcus aureus* as the model organism.

**Methods:**

The first stage of this study investigated the potential for *S. aureus* to develop tolerance to high-intensity 405 nm light if pre-cultured in low-level stress violet-blue light (≤1 mW/cm^2^) conditions. Secondly, the potential for tolerance development in bacteria subjected to repeated sub-lethal exposure was compared by carrying out 15 cycles of exposure to high-intensity 405 nm light, using a sub-lethal dose of 108 J/cm^2^. Inactivation kinetics and antibiotic susceptibility were also compared.

**Results:**

When cultured in low-level violet-blue light conditions, *S. aureus* required a greater dose of high-intensity 405 nm light for complete inactivation, however this did not increase with multiple (3) low-stress cultivations. Repeated sub-lethal exposures indicated no evidence of bacterial tolerance to 405 nm light. After 15 sub-lethal exposures 1.2 and 1.4 log_10_ reductions were achieved for MSSA and MRSA respectively, which were not significantly different to the initial 1.3 log_10_ reductions achieved (*P =* 0.242 & 0.116, respectively). Antibiotic susceptibility was unaffected, with the maximum change in zone of inhibition being *±* 2 mm.

**Conclusions:**

Repeated sub-lethal exposure of non-proliferating *S. aureus* populations did not affect the susceptibility of the organism to 405 nm light, nor to antibiotics. Culture in low-level violet-blue light prior to 405 nm light exposure may increase oxidative stress responses in *S. aureus*, however, inactivation still occurs and results demonstrate that this is unlikely to be a selective process. These results demonstrate that tolerance from repeated exposure is unlikely to occur, and further supports the potential development of 405 nm light for clinical decontamination and treatment applications.

## Background

There is global concern surrounding antibiotic resistant organisms, such as methicillin-resistant *Staphylococcus aureus* (MRSA). These organisms negatively impact on healthcare systems by causing infections which are harder to treat due to reduced antibiotic choices, resulting in longer hospital stays and increased mortality of patients [1]. To reduce transmission of these pathogenic organisms, new technologies are being developed to aid environmental decontamination and clinical treatment.

One such antimicrobial technology is 405 nm light. Violet-blue light in this region photo-excites intracellular porphyrins within microorganisms, producing a range of reactive oxygen species (ROS) which cause oxidative damage and cell death [2–4]. Although less germicidal than ultraviolet (UV) light, 405 nm light has broad-spectrum antimicrobial action against Gram positive and negative bacteria, bacterial biofilms, endospores, yeasts, fungi and in some circumstances viruses [5–11]. When used at appropriate irradiances, these wavelengths of visible light can also exert antimicrobial effects whilst being non-detrimental to mammalian cells [12–14], giving it operational advantages over UV-light for applications such as continuous decontamination of occupied environments [15–18] and wound decontamination [12]. Additionally, it has recently been reported that 405 nm light can also have a synergistic antimicrobial effect with common chlorinated disinfectants, providing further support for its beneficial use for environmental decontamination [19].

Although 405 nm light has extensive antimicrobial action and safety advantages, little is known about the potential for the development of bacterial resistance or tolerance to violet-blue 405 nm light inactivation. It is hypothesised that tolerance is unlikely due to the mechanism of inactivation [13, 17, 20]. Similar to that of photodynamic inactivation (PDI), which makes use of an additional photosensitizer, the mechanism of inactivation is thought to be non-selective, as the ROS and ^1^O_2_ produced cause unspecific damage to a wide spectrum of targets within bacterial cells [13, 17, 20, 21]. Nevertheless there is little evidence available to support this hypothesis. Several studies have investigated tolerance formation following repeated PDI in a range of microorganisms including methicillin-sensitive and methicillin-resistant *S. aureus*, *Escherichia coli*, *Pseudomonas aeruginosa, Peptostreptococcus micros*, *Actinobacillus actinomycetes* and *Vibrio fischeri*, in which none of the aforementioned species were found to become tolerant [21–25]. However little is known about the potential for bacterial tolerance development to antimicrobial 405 nm light alone.

This study was carried out to determine if there is potential for tolerance development in exposed organisms, using the nosocomial pathogen *S. aureus* as the model organism. The first stage of the study assessed whether pre-culture in low-level light-stress conditions would subsequently affect the susceptibility of the organism to 405 nm light inactivation and increase tolerance. Secondly, the study investigated the effect of repeated sub-lethal exposure to high-intensity 405 nm light on methicillin-sensitive and methicillin-resistant *S. aureus*, with the number of sub-lethal exposures being extended past those carried out in previously published studies [20, 26, 27]. To further investigate the likelihood of tolerance, the inactivation kinetics and antibiotic susceptibility of survivors after sub-lethal exposure were analysed and compared. The results provide novel fundamental evidence to support the hypothesis that tolerance following repeated exposure to 405 nm light is unlikely in both proliferating and non-proliferating bacteria.

## Methods

### Microorganisms

Two strains of *Staphylococcus aureus* were used in this study, methicillin-sensitive *S. aureus* NCTC 4135 (National Collection of Type Cultures, Collindale, UK) and epidemic methicillin-resistant *S. aureus-*15 (EMRSA-15) (Scottish Reference Laboratory, Glasgow, UK), which are referred to as MSSA and MRSA throughout the study.

### Determining potential for tolerance development when cultured under different lighting conditions

#### Cultivation in different lighting conditions

**Fig. 1:**
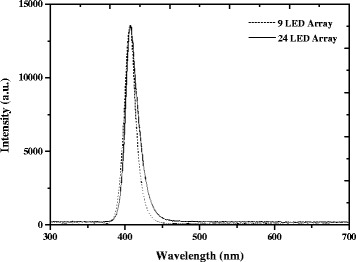
Optical emission spectrum of the 9-LED and 24-LED 405 nm arrays. Measured using an HR4000 spectrometer (Ocean Optics, Germany) and Spectra Suite software version 2.0.151

MSSA was inoculated in 100 ml nutrient broth (NB; Oxoid, UK) and cultivated under different lighting conditions: (i) complete darkness (flasks wrapped in aluminium foil); (ii) normal laboratory fluorescent lighting, approximately 200 Lux (measured using a Light Level Meter; Labfacility, UK); and (iii) 3 levels of low-intensity 405 nm light: 0.15, 0.5 & 1 mW/cm^2^ (measured using a radiant power meter and photodiode detector (LOT Oriel, USA)). The light source used was a low-power matrix of 9-light emitting diode arrays (LED) (GE Illumination, USA) with a peak wavelength in the region of 405 nm and a full width at half maximum (FWHM) of approximately 18 nm (Fig. 1). Arrays were arranged in a 3 × 3 grid pattern on a heat sink (for heat dissipation), and powered by a variable DC power supply (Velleman, Belgium). The light source and power supply were placed in the 37 °C incubator, 30 cm from the cultivation flask, and allowed to stabilise before setting the irradiance; this ensured that the current would not fluctuate during temperature increase. Utilising this light source enabled low level illumination of a large surface area (culture flask) during bacterial cultivation*.* Bacteria were cultured in these lighting conditions under rotary incubation (120 rpm) at 37 °C for 18–24 h. The broths were then centrifuged at 3939×*g* for 10 min and the pellets re-suspended in 100 ml phosphate buffer saline (PBS; Oxoid, UK). Bacterial suspensions were then diluted in PBS to an experimental starting population of 1–2 × 10^5^ CFU/ml for exposure to high-irradiance 405 nm light.

#### Exposure to high-irradiance 405 nm light following cultivation under different lighting conditions

Following cultivation in the different lighting conditions, the bacteria where exposed to high-irradiance 405 nm light to establish if their susceptibility to 405 nm light had been affected. The high-power light source used for exposure was a 24-LED array (PhotonStar Technologies, UK) with peak wavelength in the region of 405 nm and 19 nm FWHM (Fig. 1). The source was connected to a heat sink and cooling fan for heat dissipation, and was powered by a 40 V Xitanium LED Driver (Phillips, Netherlands). This light source provided high-intensity illumination over a small surface area, ideal for exposure of samples in 6-well plates.

The array was held in a PVC housing approximately 6 cm above the surface of the bacterial samples. Five millileters bacterial samples were exposed to 405 nm light at an irradiance of 60 mW/cm^2^, with sample plates positioned on a magnetic stirring plate to permit constant agitation. Control plates were held under identical conditions however only subjected to normal laboratory lighting. Samples were exposed to increasing doses of light, with dose (J/cm^2^) calculated as irradiance (W/cm^2^) × exposure time (s). During exposure, samples were taken at 15 min intervals and plated onto nutrient agar (NA; Oxoid, UK). Plates were incubated at 37 °C for 24 h and then enumerated, with results reported as colony forming units per millilitre (CFU/ml) as a function of dose (J/cm^2^).

#### Investigating the minimum inhibitory concentration of H_2_O_2_

To investigate if there were any intracellular changes in catalase production following growth in low-level violet-blue light, a minimum inhibitory concentration (MIC) assay of hydrogen peroxide (H_2_O_2_) was performed. This was based upon methodology used by Lipovsky et al. [28]. Briefly, following 4–5 h of cultivation in fluorescent laboratory lighting, complete darkness or 1 mW/cm^2^ 405 nm light, 100 μl of MSSA (10^6^ CFU/ml) was added to two-fold dilutions of H_2_O_2_ in NB. The H_2_O_2_ dilutions were then incubated at 37 °C for 24 h and the MIC identified as the lowest concentration of H_2_O_2_ which caused visible inhibition of bacterial growth.

#### Carotenoid (Staphyloxanthin) extraction

To investigate if carotenoid pigments protected *S. aureus* against singlet oxygen stress during growth in low-level violet-blue light, carotenoid content was measured. Carotenoid extraction was adapted from that used by Bartolomeu et al. [25]. MSSA was cultured in fluorescent laboratory lighting, complete darkness or 1 mW/cm^2^ 405 nm light for 24 h. After cultivation the cultures were washed thrice in sterile H_2_O, re-suspended in 5 ml 99.9% methanol, and incubated in a 55 °C water bath for 15 mins until the cells had been bleached. Following extraction, the samples were centrifuged at 8000×*g* for 10 min. The supernatant was removed and re-centrifuged at 10,000×*g* for 15 mins to ensure removal of any residual biomass. The carotenoid content was then measured by recording the absorbance at 465 nm in a quartz cuvette, using a Biomate 5 Spectrophotometer (Thermo Fischer Scientific, UK).

### Determining potential for tolerance development following repeated sub-lethal exposure to high-intensity 405 nm light

#### Repeated sub-lethal exposure

MSSA and MRSA were inoculated, in triplicate, in 100 ml NB, and cultivated at 37 °C for 18–24 h under rotary conditions (120 rpm) in the dark. Cultures were re-suspended in PBS and diluted to a 10^5^ CFU/ml population. Bacterial samples were then exposed to 405 nm light at a dose of 108 J/cm^2^ (60 mW/cm^2^ for 30 min) using the high-intensity 24-LED array (as described in earlier section ‘Exposure to High-Irradiance 405 nm Light Following Cultivation under Different Lighting Conditions’).

**Fig. 2:**
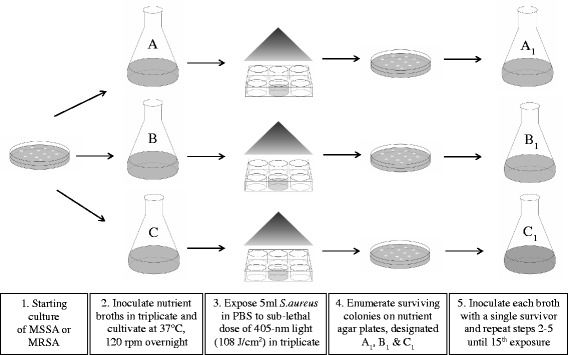
Flow diagram of the experimental procedure used during repeated sub-lethal 405 nm light exposure. Methicillin-sensitive *Staphylococcus aureus* (MSSA) and methicillin-resistant *Staphylococcus aureus* (MRSA) were exposed to 15 cycles of a dose of 108 J/cm^2^, at an irradiance of 60 mW/cm^2^

Following exposure, samples were plated onto NA and incubated at 37 °C for 24 h. Surviving colonies were enumerated and termed as survivors from ‘Run 1’. Surviving colonies from ‘Run 1’ were then used to inoculate NB, in triplicate, and the above process of exposure-subculture-exposure was repeated until 15 sub-lethal exposures had occurred (Fig. 2). Survivors from each run were tested using Staphaurex Plus Latex Agglutination (Thermo Fischer Scientific, UK) and Penicillin Binding Protein (PBP2’) Latex (Oxoid, UK) to ensure no contamination had occurred. This was based on the principle that if the Staphaurex test gave a negative result, then this would indicate that the surviving isolate was not the test organism and that contamination by a rogue organism had occurred. If however, the isolate gave a positive Staphaurex result but a negative PBP2’ result, then this would indicate that the MRSA cultures were likely contaminated with MSSA or that MRSA had lost the ability to produce PBP2’ following exposure to 405 nm light.

#### Inactivation kinetics of survivors following sub-lethal exposure

Full inactivation kinetics were also established out using the initial cultures and survivors after 5, 10 and 15 sub-lethal exposures. These isolates were cultivated in 100 ml NB in the dark and exposed to 60 mW/cm^2^ 405 nm light, with samples taken every 15 min for 75 mins and 90 mins for MSSA and MRSA respectively (as described in earlier section ‘Exposure to High-Irradiance 405 nm Light Following Cultivation under Different Lighting Conditions’).

#### Antibiotic susceptibility testing

Antibiotic susceptibility testing was carried out following the EUCAST guidelines [29] as closely as possible (variations included the use of 90 mm plates, stacking during incubation, and inoculation with only one antibiotic disc/plate to prevent overlap if bacterial susceptibility increased). The antibiotics used were chloramphenicol, ciprofloxacin, erythromycin, fusidic acid, gentamicin, mupirocin, oxacillin, rifampicin, tetracycline and vancomycin. These 10 were selected as they represented antibiotics, with differing antimicrobial mechanisms, which are recommended clinical treatment options for *Staphylococcus aureus* infections. Fresh bacterial cultures were diluted to a 10^8^ CFU/ml population, spread onto mueller-hinton (MH) agar plates (Oxoid, UK) using sterile swabs and a single antibiotic disc (Mast Group, UK) placed into the centre of the plate. The plates were immediately incubated at 35 ± 2 °C for 18–20 h after which the diameter of the zone of inhibition was measured in normal laboratory lighting and recorded to the nearest mm.

### Data analysis

Data represents mean results ± standard deviation (SD), taken from triplicate independent experiments, measured in at least duplicate for 405 nm light inactivation experiments (*n* ≥ 6). As samples were directly plated onto NA plates, some samples counted were below the detection limit (25 CFU/ml) however these have been included in graphs to demonstrate the near-to-complete inactivation effect achieved. Repeated sub-lethal exposure results are reported as bacterial inactivation efficiency (log_10_ *N*_*0*_ */N*), with *N* representing the light-exposed population, and *N*_*0*_ the equivalent non-exposed control population. Significant differences were calculated using Two-Sample T-Tests or One-way ANOVA with Dunnett’s post-hoc test (Minitab 17 Statistical Software), with results found to be significant when *P* ≤ 0.05.

## Results

### Determining potential for tolerance development when cultured under different lighting conditions

#### Bacterial inactivation kinetics following single culture

**Fig. 3:**
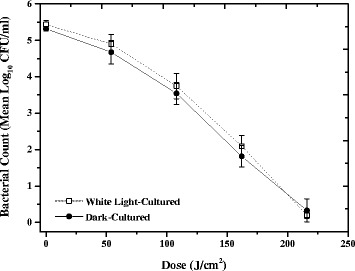
Inactivation of MSSA following culture in white light or complete darkness. Methicillin-sensitive *Staphylococcus aureus* (MSSA) was inactivated following exposure to 60 mW/cm^2^ 405 nm light after culture in fluorescent laboratory lighting (white light) or complete darkness. Each data point is a mean value ± SD (*n* ≥ 6). No significant differences were found between the susceptibility of the light and dark cultured MSSA, using two-sample t-Test (*P* ≥ 0.05). No significant changes were observed in the final control populations (*P* ≥ 0.05)

The first stage of the study investigated 405 nm light susceptibility of methicillin-sensitive *S. aureus* (MSSA) following growth in different lighting conditions. As shown in Fig. 3, relatively linear inactivation kinetics were demonstrated for both MSSA cultivated in fluorescent laboratory light (white light) and in complete darkness, with 5 log_10_ reductions achieved after a final dose of 216 J/cm^2^ 405 nm light. No significant differences in inactivation between MSSA cultures cultivated in fluorescent laboratory light or in complete darkness were detected at any of the tested sampling points using a Two-Sample t-Test (*P* ≥ 0.05). No significant change was seen in the equivalent non-exposed controls (*P* = 0.932 & 0.747 for light and dark cultures, respectively).

**Fig. 4:**
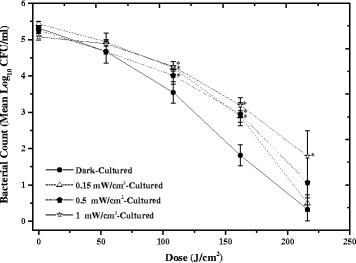
Inactivation of MSSA following overnight culture in low-level 405 nm light. Methicillin-sensitive *Staphylococcus aureus* (MSSA) was inactivated following exposure to 60 mW/cm^2^ 405 nm light after overnight culture in low-level 405 nm light (0.15, 0.5 and 1 mW/cm^2^). The inactivation kinetics of dark-cultured MSSA have been included for reference. Each data point is a mean value ± SD (n ≥ 6). * Indicates 405 nm light-cultured samples that were significantly different to those which had been dark-cultured, using one way ANOVA with Dunnett’s post-hoc test (*P* < 0.05). No significant changes were observed in the final control populations (*P* ≥ 0.05)

When cultured under low-intensity 405 nm light, some slight differences in the inactivation kinetics were observed. Although trends appeared similar (Fig. 4), differences in the susceptibility of bacteria cultured in low-intensity 405 nm light versus dark cultured can be seen at certain points during the experiment. Following exposure to 108 and 162 J/cm^2^ high-intensity 405 nm light, MSSA cultured in 0.15, 0.5 and 1 mW/cm^2^ 405 nm light showed significantly less inactivation than that cultured in complete darkness (*P* < 0.05). By a final dose of 216 J/cm^2^, the inactivation achieved between the dark cultured and low-irradiance 405 nm light cultured MSSA was similar (~4–5 log_10_ reduction), with the exception of the MSSA cultured in 1 mW/cm^2^ 405 nm light: when cultured under this lighting condition, only 3.3 log_10_ reduction was achieved compared to 5 log_10_ reduction for dark-cultivated MSSA (*P* = 0.045). No significant change was seen in the equivalent non-exposed controls (*P* = 1.00, 0.268 & 0.744 for 0.15, 0.5 and 1 mW/cm^2^ cultures, respectively).

#### Investigating the minimum inhibitory concentration of H_2_O_2_

Table 1 shows that the average MIC of H_2_O_2_ is significantly higher for MSSA cultured in 1 mW/cm^2^ 405 nm light (*P =* 0.011) compared to when cultured in white light (*P* = 0.044) or complete darkness (*P* = 0.024).

**Table 1:** Minimum Inhibitory Concentration of H_2_O_2_ and carotenoid content of *Staphylococcus aureus* following different culture conditions

Growth Conditions	Average Minimum Inhibitory Concentration of H_2_O_2_ (%) ± SD	Average Carotenoid Absorbance at 465 nm ± SD
Complete Darkness	0.00293 ± 0.00000	0.90 ± 0.22
White Lighting	0.00513 ± 0.00146	0.82 ± 0.24
Low irradiance (1 mW/cm^2^) 405 nm Light	0.01831 ± 0.01025^*^	0.17 ± 0.12^*^

Differences in the minimum inhibitory concentration (MIC) of hydrogen peroxide (H_2_O_2_) and carotenoid content of methicillin-sensitive *Staphylococcus aureus* when cultivated in different lighting conditions. Data points represent the mean count (*n* = 3) ± SD

^*^represents a significant change (*P <* 0.05)

#### Carotenoid (Staphyloxanthin) extraction

Results shown in Table 1 demonstrate that the presence of the carotenoid staphyloxanthin is significantly lower in MSSA cultured in 1 mW/cm^2^ 405 nm light (*P =* 0.009) compared to that grown in white light (*P* = 0.015) or complete darkness (*P* = 0.008).

#### Bacterial inactivation kinetics following multiple cultures

**Fig. 5:**
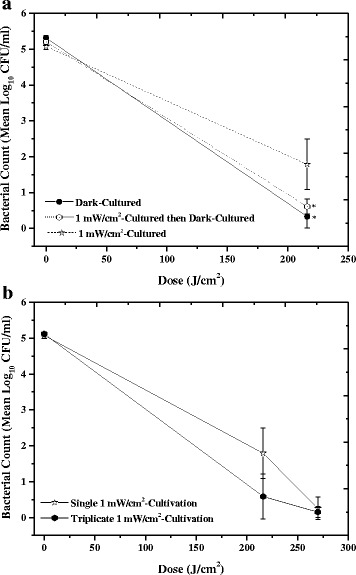
Comparison of MSSA susceptibility to high-intensity 405 nm light after cultivation in different lighting conditions. **a** Methicillin-sensitive *Staphylococcus aureus* (MSSA) was cultivated in low-intensity (1 mW/cm^2^) 405 nm light followed by cultivation in complete darkness, and **b** MSSA was subjected to repeated (three times) cultivation in low-intensity (1 mW/cm^2^) 405 nm light. Each cultivation was at 37 °C for 18 h. Following these cultivation conditions, bacteria were exposed to increasing doses of high-intensity 60 mW/cm^2^ light to establish the inactivation kinetics. Inactivation kinetics of bacteria which had been pre-cultured once in darkness or low-intensity 405 nm light are given as a comparison. * Indicates cultured samples that were significantly different to those which had been cultured once in 1 mW/cm^2^ 405 nm light, using one way ANOVA with Dunnett’s post-hoc test (*P* < 0.05). Each data point is a mean value ± SD (n ≥ 6). No significant changes were observed in the final control populations (*P* ≥ 0.05)

MSSA was grown in 1 mW/cm^2^ 405 nm light, sub-cultured onto NA plates, then re-cultured in (a) complete darkness once or (b) 1 mW/cm^2^ a further two more times. As can be seen in Fig. 5a, when cultivated in 1 mW/cm^2^ followed by cultivation in complete darkness, the level of inactivation returns to that of cultivation in darkness alone, with no significant difference in the level of inactivation (*P =* 0.395), and similarly the level of inactivation was significantly greater than that of MSSA grown in 1 mW/cm^2^ 405 nm light (*P =* 0.043). No significant change was seen in the equivalent non-exposed controls (*P* = 0.505). Figure 5b also demonstrates that triplicate cultivation in 1 mW/cm^2^ does not select for more tolerant cultures. The sensitivity of MSSA to high-intensity 405 nm light returns to a similar level of that when cultivated in complete darkness alone, with an average 0.7 × 10^1^ CFU/ml population surviving following a dose of 216 J/cm^2^, which is not significantly different to the average 0.3 × 10^1^ CFU/ml surviving population which had been grown in darkness (*P* = 0.230). No significant change was seen in the equivalent non-exposed controls (*P* = 0.338).

### Determining potential for tolerance development following repeated sub-lethal exposure to high-intensity 405 nm light

#### Repeated sub-lethal exposure

**Fig. 6:**
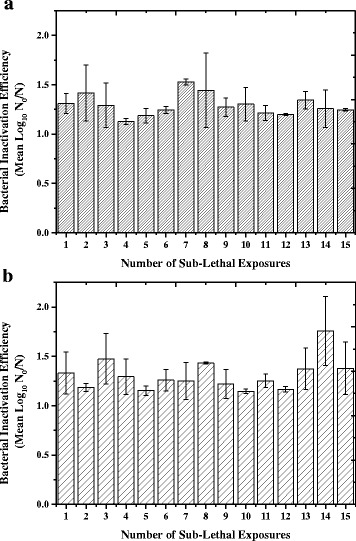
Average 405 nm light inactivation efficiency following repeated sub-lethal exposure of MSSA and MRSA. 405 nm light inactivation efficiency of 15 sub-lethal exposure cycles of (**a**) methicillin-sensitive *Staphylococcus aureus* (MSSA) and (**b**) methicillin-resistant *Staphylococcus aureus* (MRSA), to a dose of 108 J/cm^2^ 405 nm light at an irradiance of 60 mW/cm^2^. Repeated sub-lethal exposure results are reported as bacterial inactivation efficiency (log_10_ *N*_*0*_ */N*), with *N* representing the light-exposed population, and *N*_*0*_ the equivalent non-exposed control population. Each data point is a mean value ± SD (n ≥ 6). No significant changes were observed in the test or final control populations, using one way ANOVA with Dunnett’s post-hoc test (*P* ≥ 0.05)

The next stage of this study was to investigate the likelihood of tolerance development when non-proliferating *Staphylococcus aureus* was repeatedly exposed to a sub-lethal dose of high-intensity 405 nm light A dose of 108 J/cm^2^ was selected for use, as this had been shown to cause approximately 98% (1.6–1.8 log_10_) inactivation of the organism (Figs. 3 and 4). As can be seen from Fig. 6a, after repeated sub-lethal exposure there were fluctuations in MSSA inactivation, with the maximum inactivation seen after 7 sub-lethal exposures (1.5 log_10_ reduction) and minimum after 4 sub-lethal exposures (1.1 log_10_ reduction). However there was no significant difference (*P* = 0.242) in MSSA inactivation, compared to equivalent non-exposed controls, after 1 sub-lethal exposure compared to those after 15 sub-lethal exposures, with 1.3 log_10_ and 1.2 log_10_ inactivation achieved respectively. Additionally, one-way ANOVA and Dunnett’s post-hoc analysis, using Run 1 as the control group, indicated that there was no significant difference in the bactericidal efficiency of 405 nm light (*log*_*10*_ *N*_*0*_ */N)* between the sub-lethal exposures. No significant change was seen in the equivalent non-exposed controls (*P =* 0.198).

As a comparison a clinical isolate of MRSA was also repeatedly exposed to sub-lethal levels of 405 nm light. Figure 6b demonstrates that similarly to MSSA, there were fluctuations in inactivation of MRSA, with a maximum inactivation after 14 sub-lethal exposures (1.8 log_10_ reduction) and minimum after 10 sub-lethal exposures (1.1 log_10_ reduction). Furthermore there was also no significant difference (*P* = 0.943) between the 1.3 log_10_ and 1.4 log_10_ reduction after 1 and 15 sub-lethal exposures respectively. Similarly to MSSA, one-way ANOVA and Dunnett’s post-hoc analysis, using Run 1 as the control group, indicated that there was no significant difference in the bactericidal efficiency of 405 nm light (*log*_*10*_ *N*_*0*_ */N)* between the sub-lethal exposures. No significant change was seen in the equivalent non-exposed controls (*P =* 0.116).

#### Inactivation kinetics of survivors following sub-lethal exposure

**Fig. 7:**
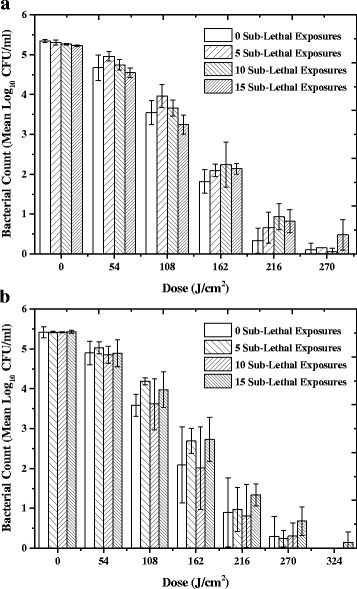
Average inactivation kinetics of MSSA and MSSA after repeated sub-lethal exposures of 405 nm light. Inactivation kinetics of isolates of (**a**) methicillin-sensitive *Staphylococcus aureus* (MSSA) and (**b**) methicillin-resistant *Staphylococcus aureus* (MRSA) after 5, 10 and 15 sub-lethal exposures of 405 nm light. Sub-lethal exposures were conducted using a dose of 108 J/cm^2^ (at an irradiance of 60 mW/cm^2^), and inactivation kinetics were based on exposure to 60 mW/cm^2^ for increasing time periods. Results are compared to non-sub-lethally exposed bacteria as a control. Each data point is a mean value ± SD (n ≥ 6). No significant changes were observed in the test populations, using one way ANOVA with Dunnett’s post-hoc test (*P* ≥ 0.05)

The inactivation kinetics of surviving MSSA and MRSA isolates after 5, 10 and 15 sub-lethal exposures to 60 mW/cm^2^ light were also determined. Figure 7a shows that after a dose of 270 J/cm^2^ (75 mins) there was no significant difference in the level of MSSA inactivation after 5, 10 and 15 sub-lethal exposures, with 5.2 (*P =* 0.895), 5.2 (*P =* 0.795) and 4.8 (*P =* 0.583) log_10_ reduction achieved, compared to non-sub-lethally exposed MSSA. Similarly, MRSA showed no significant difference in inactivation after a dose of 324 J/cm^2^ (90 mins) with 5.4 (*P* = 0.193), 5.4 (*P* = 0.259) and 5.3 (*P* = 0.081) log_10_ reductions of MRSA after 5, 10 and 15 sub-lethal exposures, respectively.

#### Antibiotic susceptibility testing

Although little change was seen in bacterial susceptibility to high-intensity 405 nm light after repeated sub-lethal exposure, antibiotic susceptibility was also investigated.

Table 2 shows that there was little variation in the diameter of the zones of inhibition for MSSA after 5, 10 and 15 sub-lethal exposures compared to the initial non sub-lethally exposed populations. There were no significant decreases in the diameter of the zone of inhibition between the initial non sub-lethally exposed MSSA and survivors of 15 sub-lethal exposures (*P* > 0.05). There was a slight decrease in the average diameter of the zone of inhibition caused by mupirocin after 5 sub-lethal exposures, however after 10 and 15 sub-lethal exposures inhibition was comparable to non-stressed cultures (*P* > 0.05).

**Table 2:** Antibiotic susceptibility of MSSA and MRSA following sub-lethal exposure to 405 nm light

Antibiotic	Conc (μg)	Mean Diameter of Zone of Inhibition of MSSA (mm)				Mean Diameter of Zone of Inhibition of MRSA (mm)			
		0	5	10	15	0	5	10	15
Chloramphenicol	25^a^	21 ± 1.5	21 ± 0.8	21 ± 1.2	21 ± 0.8	24 ± 1.0	24 ± 1.1	23 ± 1.4	23 ± 1.1
Ciprofloxacin	5	22 ± 1.2	21 ± 0.5	22 ± 0.7	22 ± 1.3	26 ± 0.6	26 ± 0.4	26 ± 1.0	26 ± 1.1
Erythromycin	5^a^	21 ± 0.6	21 ± 0.6	22 ± 0.8	21 ± 0.4	23 ± 1.0	22 ± 0.8	23 ± 1.0	23 ± 1.2
Fusidic Acid	10	30 ± 0.0	30 ± 0.8	32 ± 1.0*	31 ± 1.8	31 ± 0.6	30 ± 0.5	30 ± 0.7	30 ± 0.9
Gentamicin	10	21 ± 0.6	21 ± 0.5	21 ± 0.5	20 ± 0.5	21 ± 0.6	21 ± 0.5	22 ± 1.3	23 ± 0.6*
Mupirocin	5^a^	28 ± 0.6	26 ± 0.5*	28 ± 1.4	28 ± 1.1	26 ± 0.6	27 ± 1.1	26 ± 1.2	26 ± 1.3
Oxacillin	5^b^	30 ± 0.0	29 ± 0.9	30 ± 0.6	30 ± 0.9	0 ± 0.0	0 ± 0.0	0 ± 0.0	0 ± 0.0
Rifampicin	5	32 ± 1.0	32 ± 0.7	33 ± 1.8	34 ± 0.6*	31 ± 0.6	30 ± 0.7	31 ± 1.0	31 ± 0.9
Tetracycline	25^a^	28 ± 1.2	28 ± 1.0	28 ± 1.4	29 ± 1.3	27 ± 1.0	27 ± 0.7	27 ± 1.0	26 ± 0.9
Vancomycin	5^b^	15 ± 0.0	14 ± 0.7	15 ± 0.7	15 ± 0.5	14 ± 0.6	15 ± 0.6	15 ± 0.5	14 ± 0.5

Antibiotic susceptibility of methicillin-sensitive *Staphylococcus aureus* (MSSA) and methicillin-resistant *Staphylococcus aureus* (MRSA) after 5, 10 and 15 sub-lethal exposures compared to equivalent non-sub-lethally exposed controls (0). Susceptibility was measured using Disc Diffusion Method

*indicates diameters of zones of inhibition that was significantly different to the equivalent non-sub-lethally exposed control (*P* ≤ 0.05). Each data point is a mean value ± SD (*n* ≥ 3)

^a^Disc concentration is lower than that recommended by EUCAST

^b^No disc concentration recommended by EUCAST

With regards to the antibiotic susceptibility of MRSA isolates after sub-lethal exposure, there were no significant decreases between the initial non-sub-lethally exposed populations and those after 15 sub-lethal exposures.

Interestingly there were significant increases in the diameter of the zone of inhibition for fusidic acid after 10 sub-lethal exposures and rifampicin after 15 sub-lethal exposures for MSSA and with gentamicin after 15 sub-lethal exposures for MRSA, compared to initial non sub-lethally exposed populations.

## Discussion

This study has addressed the question of the potential for *S. aureus* to become tolerant to antimicrobial 405 nm violet-blue light and has provided new information under carefully controlled experimental test conditions.

The results from the first stage of the study, demonstrated that MSSA cultured in 1 mW/cm^2^ 405 nm light appeared to exhibit a degree of tolerance to high-intensity 405 nm light, compared to when dark-cultured. This suggests that exposure to low-intensity 405 nm light during culture may have acted as a low-level stressor during growth, which could have increased the up-regulation of bacterial oxidative stress responses. Bacteria have developed several mechanisms to overcome oxidative stress, including enzymes to detoxify reactive oxygen species such as catalase, peroxidase and superoxide dismutase [30]. To detect changes in the ability of MSSA to tolerate oxidative stress after culture in the presence of 1 mW/cm^2^ 405 nm light, compared to white light or complete darkness, a MIC assay using H_2_O_2_ was carried out, and carotenoid content was measured. Results determined that the average MIC of H_2_O_2_ was significantly higher for the organism when cultured in low-level (1 mW/cm^2^) 405 nm light compared to white light or complete darkness. This demonstrates that during growth in low-irradiance 405 nm light there is likely an increase in the expression of protective enzymes e.g. an increase in catalase enzymes which would act as hydrogen peroxide scavengers. An increase in catalase enzyme, KatA, following photodynamic inactivation of *S. aureus* (using blue light and exogenous porphyrins) was also demonstrated by Doselli et al. [31], which further suggest this enzyme is up-regulated to try to help protect the bacteria against oxidative stress.

The presence of the carotenoid staphyloxanthin was also found to be significantly lower in MSSA cultured in low-level (1 mW/cm^2^) 405 nm light compared to when grown in white light or darkness. As carotenoid pigments have anti-oxidative properties, in particular for protecting bacteria against ^1^O_2_ stress [32], you would expect MSSA cultivated in 1 mW/cm^2^ 405 nm light to be more sensitive to subsequent high-intensity 405 nm light exposure, due to the lower levels of staphyloxanthin present. However, as tolerance is increased upon exposure to high-intensity 405 nm light, it is likely there is an up-regulation of other oxidative stress responses within the bacteria and that the carotenoid pigment provided protection against ROS during growth in low-intensity 405 nm light. Further experiments investigating superoxide dismutase, catalase activity could help to confirm this.

Although the inactivation achieved at 216 J/cm^2^ (Fig. 4) was less when the bacteria had been pre-cultured in low-irradiance (1 mW/cm^2^) 405 nm light, it is important to note that when exposed to a higher dose of 270 J/cm^2^ complete 5 log_10_ inactivation was still able to be achieved (Fig. 5b). It is likely that complete inactivation is still able to occur due the level of ROS produced being greater than the level that the basal bacterial oxidative stress defence systems are able to scavenge [30].

It is important to consider the possibility that bacteria may become less sensitive to high-irradiance 405 nm light after pre-culture in low-intensity, sub-lethal stress levels of 405 nm light, as this could have implications when violet-blue light is used to inactivate bacteria in nutritious environments where the bacteria are able to replicate, for example within wounds or surgical sites. In these cases it would be important to ensure a bactericidal dose was administered: if the dose administered or irradiance used is too low, the bacteria may not be completely inactivated and cause pathogenic organisms to potentially become more tolerant to subsequent applications of violet-blue light. Additionally, if the dose delivered is too low there may actually be an increase in population concentration, as it is thought visible light can encourage proliferation of microorganisms when used on nutrient rich areas such as wounds [28].

Further investigations were carried out to investigate if growth in low-intensity 405 nm light was selective for MSSA which was able to adapt to a greater level of oxidative stress. Results demonstrated that this was not the case, with the sensitivity of MSSA returning to a similar level of that when cultivated in complete darkness alone. These results additionally suggest that the increased tolerance to high-intensity 405 nm light is caused by an up-regulation in bacterial stress response rather than selection for violet-blue light tolerant colonies due to growth conditions.

Further work should be carried out to investigate the oxidative stress response following growth in low-intensity 405 nm light fully, such as the levels of superoxide dismutase, which has been previously shown to be up-regulated in *S. aureus* sensitive to PDI [33]. Additionally it is known that there is a heat shock protein cascade after PDI [30] and a study by St Denis et al. [34] demonstrated that *E. coli* exposed to external stress, before PDI showed a 2 log_10_ less reduction in bacterial inactivation compared to normal PDI inactivation levels. Therefore it would be interesting to investigate if there was a heat shock protein cascade during growth in 1 mW/cm^2^ violet-blue light, and if this up-regulation of proteins before high-intensity exposure may contribute to the stress tolerance seen. Additionally, investigations should explore why the increased level of tolerance is not seen after repeated growth in the low-level stress conditions. However as the bacteria can still be completely inactivated, and as results indicated repeated growth in low-irradiance light is unlikely to be a selective process, MSSA should continue to be susceptible to high-irradiance 405 nm light after growth in the presence of a low-level stressor.

It is also important to note that the changes in susceptibility observed here are in response to the organisms being pre-cultured in low-level 405 nm light. This is unlikely to occur in non-proliferating bacteria, such as is the case with environmental contamination [17], as these organisms are stressed and not actively growing, thus up-regulation of stress responses is unlikely to occur, and actually when in a stationary stressed state, bacteria should become more susceptible to 405 nm light inactivation [35].

The next stage of this study was to investigate the likelihood of tolerance development when non-proliferating bacteria were repeatedly exposed to a sub-lethal dose of high-intensity 405 nm light. MSSA and MRSA were subjected to 15 exposure-subculture-exposure cycles to 60 mW/cm^2^ 405 nm light, resulting in a dose of 108 J/cm^2^ per sub-lethal exposure. Results demonstrated no significant change in the level of inactivation achieved, with 1.2 log_10_ and 1.4 log_10_ inactivation achieved for MSSA and MRSA after 15 sub-lethal exposures respectively, compared to an initial 1.3 log_10_ inactivation achieved for both.

The dose of 108 J/cm^2^ was selected for exposure as it was shown to cause approximately 98% (1.6–1.8 log_10_) inactivation of the organism used in this study (Figs. 3 and 4). When compared to doses used in other studies to achieve similar levels of inactivation, this dose is somewhat greater [6, 7], however, the dose dependency will vary between studies, as it is specific to the light sources, the irradiances and the organisms used. LED arrays have variations in spectral output in terms of exact peak wavelength and bandwidth, and therefore this will affect the efficiency of bacterial inactivation. In addition, the overall doses used will vary depending on the output irradiance of the light source: the higher power the light source, the higher the irradiance, however, given that there will be a finite number of porphyrins per bacterial cell capable of photon absorption, there is likely to be a point at which the absorption of more photons would have little effect on the inactivation mechanism already in progress, therefore although more energy is being delivered, it may not translate into more effective kill.

Little other evidence has been documented regarding bacterial tolerance to 405 nm light. A short study by Guffey et al. [26] demonstrated that there was potential for *S. aureus* to become tolerant to a dose of 9 J/cm^2^ 405 nm light, as following 5 repeated exposures there was a decrease in the kill rate seen. The study demonstrated an initial increase in kill rate from 32.92 to 59.49% after the 1st to 4th sub-lethal exposures, however kill rate subsequently declined, with only 18.04% kill after the 7th repeated sub-lethal exposure, attributed to the development of resistance. These results are conflicting with those in this study, as our results indicate little change in bacterial response to 405 nm light inactivation following 15 sub-lethal exposures. In the present study, which used more than double the number of sub-lethal exposures and higher bacterial populations, natural variation in the level of inactivation occurred after repeated sub-lethal exposure, and this variation would be particularly apparent when using low population densities, as was the case in the study by Guffey et al. [26], so potentially further repeated sub-lethal exposures may have shown a subsequent increase in kill rate.

Additionally, in the present study *S. aureus* was repeatedly sub-lethally exposed whilst suspended in PBS. This allowed investigation of the sole effect that repeated antimicrobial violet-blue light exposure would have on bacterial cultures in a stationary, non-proliferating, state, representative of how bacteria would be found in the clinical environment. In the study by Guffey et al. [26], *S. aureus* was exposed to 405 nm light whilst seeded onto mannitol salt agar plates. Not only is mannitol known to be a ROS scavenger [36], in this scenario the bacteria were likely to be in a metabolic state, tolerating the high salt conditions (7.5% NaCl) and fermenting mannitol, and therefore these processes may have affected the subsequent bacterial stress response to violet-blue light. Consequently the increased tolerance seen may be more relative to results in the earlier phase of this study, where low-level exposure to 405 nm light in nutritious conditions resulted in higher dose requirements for complete inactivation.

Conversely, two studies which exposed *Acinetobacter baumannii* and *P. aeruginosa* to 415 nm light, under exposure conditions similar to this study, demonstrated no evidence of tolerance formation [20, 27]. Results are in keeping with study, with no tolerance to 415 nm light inactivation seen in 10^8^ CFU/ml populations of *P. aeruginosa* and *A. baumannii* after 10 repeated sub-lethal exposures to a dose of 36 J/cm^2^ and 70.2 J/cm^2^ respectively [20, 27]. These previous studies, along with the results in the present study, support the hypothesis that due to the unspecific mechanism of action and broad spectrum of intracellular targets, tolerance is not likely to occur.

Interestingly in the study by Zhang et al. [27] a significant increase in the sensitivity of *A. baumannii* was seen between the 1st exposure (4.52 log_10_ reduction) and the 10th exposure (6.28 log_10_ reduction) and inactivation curves revealed an increase in inactivation between 1, 6 and 9 sub-lethal exposures. These results were thought to indicate that a favourable mutation had occurred, increasing bacterial susceptibly to violet-blue light inactivation [27]. To investigate if this phenomenon would also occur in *Staphylococcus aureus*, the inactivation kinetics of surviving isolates after 5, 10 and 15 sub-lethal exposures were determined and all followed similar trends. Although these results do not indicate an increase in sensitivity of MSSA and MRSA to 405 nm light after repeated sub-lethal exposure, they do demonstrate consistent *Staphylococcal* sensitivity to high-irradiance 405 nm light and further supports the hypothesis that tolerance to 405 nm light inactivation in unlikely. However, the potential for tolerance should be evaluated in other microorganisms normally susceptible to 405 nm light inactivation, including multidrug resistant organisms which are currently a great problem in healthcare settings [37, 38].

Antibiotic susceptibility was also analysed to ensure that repeated 405 nm light exposure did not give rise to ‘stress hardening’, whereby as a result of continued exposure to this sub-lethal stress the bacteria would be able to adapt and develop protection mechanisms against other applied stresses [39, 40]. Little significant variation was seen with the antibiotic susceptibility for both *S. aureus* strains. Although a slight decrease in susceptibility of MSSA to mupirocin was noted after 5 sub-lethal exposures (an observation which was not observed following increased sub-lethal exposures to 10 or 15 cycles), it is worth noting that although the concentration of mupirocin antibiotic disc (5 μg) was below the recommended concentration used by EUCAST (200 μg), the zone of inhibition measured was still far greater (26 mm) than the EUCAST breakpoints for resistance (18 mm). Therefore, indicating that although significantly different to the initial non sub-lethally exposed MSSA, after 5 sub-lethal exposures MSSA is bacteria is still sensitive to mupirocin.

Furthermore, to the best of our knowledge these are the first results comparing repeated sub-lethal 405 nm light exposure and antibiotic susceptibility, and they indicate that sub-lethal exposure is unlikely to result in the development of antibiotic resistance. These findings are further supported by those of Pedigo et al. [23], who demonstrated no antibiotic resistance occurred in MSSA after 25 repeat exposures to PDI, using a methylene blue photosensitizer and 670 nm light, compared to antibiotic resistance in MSSA after only 11 exposures to a 1 μg/ml oxacillin disk. Similarly, Grinholc et al. [41] found no change in antibiotic susceptibility in MRSA before and after exposure to PDI (using protoporphyrin diarginate and 624 nm light) with the 26 different antibiotics tested.

Future studies should involve a greater range of commonly used antibiotics and broth microdilution studies should be carried out to enable the quantification of the MIC of each antibiotic before and after sub-lethal exposure. However, overall these results indicate repeated sub-lethal exposure to 405 nm light is unlikely to affect *S. aureus* susceptibility to antibiotics, and supports the practical application of 405 nm light for decontamination applications within in the clinical environment [42].

This study was designed to generate fundamental microbiological information with regards to the potential for the development of bacterial tolerance to 405 nm light. To investigate this, *S. aureus* was exposed to high-irradiance light whilst in suspension, following either growth in low-level blue light stress conditions or previous sub-lethal exposures to 405 nm light. As discussed earlier, the 405 nm light levels used in the present study permitted establishment of the fundamental responses of the light-exposed bacteria. There is however, interest in utilising this antimicrobial technology for practical decontamination applications and it will therefore be important for future studies to progress investigations towards more clinically-relevant scenarios. This could include repeated exposure of bacteria on inert surfaces with low-irradiance 405 nm light more representative of levels which would be used for environmental decontamination (<1 mW/cm^2^) [15–18], or repeated exposure of organisms on tissue models using higher irradiance light, more suited for representation of wound treatments [12]. It would also be important to investigate if there are any differences when bacteria are dried onto clinical surfaces compared to when suspended in biological fluids such as blood and secretions. A previous study by Murdoch et al. [43], also demonstrated that bacteria can be more susceptible to inactivation when exposed whilst dried onto surfaces and in a desiccated state, and it is reasonable to consider that this increased susceptibility when on surfaces is likely to reduce the likelihood of persistent survival and tolerance/resistance. In addition, if the bacteria are present in biological fluids or embedded in a biological matrix then the inactivation effect could be enhanced by the excitation of photosensitive components present in the biological fluids or matrix (which accelerates the production of ROS) [10, 11]. Conversely it must also be considered that inactivation efficacy of the 405 nm light could be hindered by the opaque transmission properties of the suspending fluid/matrix. Clearly much more research is required to understand not only the tolerance response of bacteria to 405 nm light but also the complex interplay between physico-chemical and environmental factors that can impact on the efficacy of the light induced inactivation in real clinical settings.

Finally, it would also be important to establish the influence of strain variance on the potential for tolerance. Although comparisons were made between methicillin sensitive and resistant strains of *S. aureus* other studies have demonstrated that photodynamic inactivation and violet-light exposure can vary between exposed strains of the same organisms [28, 44].

## Conclusions

In conclusion, the results in this study provide novel fundamental information surrounding the potential for tolerance development in proliferating *S. aureus* cultivated in low-level 405 nm light. Additionally no evidence of tolerance was generated in non-proliferating antibiotic sensitive and resistant organisms, following repeated sub-lethal exposure. Repeated sub-lethal exposure was also shown to have little effect on inactivation kinetics or antibiotic susceptibility. Whilst previous studies have established that violet-blue light can be safely used for continuous decontamination in occupied environments [15, 16, 18], wound decontamination in mouse models [13, 20, 27, 45], and that it can inactivate a wide range of microorganisms [5–11], our current findings indicate that in fundamental studies using high-intensity light irradiances, bacterial tolerance is unlikely to occur even after repeated use.

These proof-of-principle results offer additional evidence towards the effective use of antimicrobial 405 nm light for decontamination applications within the clinical setting, however further work is needed to translate these findings to use lower, more clinically-relevant irradiance levels and/or longer duration exposures, relevant for applications such as environmental and wound decontamination.

## Abbreviations

^1^O_2_: Singlet oxygen
CFU: Colony forming units
EMRSA – 15/MRSA: Epidemic methicillin-sensitive *Staphylococcus aureus*-15
FHWM: Full width at half maximum
H_2_O_2_: Hydrogen peroxide
LED: Light emitting diode
MH: Mueller-hinton
MIC: Minimum inhibitory concentration
MSSA: Methicillin-sensitive *Staphylococcus aureus*
NA: Nutrient agar
NB: Nutrient broth
PBS: Phosphate buffered saline
PDI: Photodynamic inactivation
ROS: Reactive oxygen species
SD: Standard deviation
UV: Ultraviolet
